# Factors determining the social participation of older adults: A comparison between Japan and Korea using EASS 2012

**DOI:** 10.1371/journal.pone.0194703

**Published:** 2018-04-06

**Authors:** Keiko Katagiri, Ju-Hyun Kim

**Affiliations:** 1 The Graduate School of Human Development and Environment, Kobe University, Kobe, Hyogo Prefecture, Japan; 2 Department of Sociology, Chungnam National University, Daejeon, South Korea; Ehime University Graduate School of Medicine, JAPAN

## Abstract

**Aims:**

Japan and Korea are the world’s most aged and most rapidly aging nations. They both have low fertility rates, thereby intensifying the importance of social structures to aid a large, dependent population of older adults. Common strategies involve improving their social participation, which enhances their physical and mental health, so they are supporting society rather than being supported. Since the social participation rates in both countries are not as high as those of Western countries, it is critical to shed light on the factors related to social participation of the elderly.

**Methods:**

A secondary analyses were performed using Japanese and Korean data from the 2012 East Asia Social Survey (EASS), which includes nationally representative samples through random sampling. The analyses only include data from those 65 and older (Japan: *N* = 683, Korea: *N* = 362).

**Results:**

Social participation is classified into four types: 1) *no affiliation*; 2) *inactive participation*; 3) *active recreational*; and 4) *active social*. The Japanese respondents had a higher participation rate than Koreans, but more Japanese were *inactive*. Though the rates of *active* participations were similar in both countries. Multinomial logistic regressions were conducted to examine the related factors among the four types of social participation. Basic attributes (e.g., living alone) and other factors (e.g., network size) were included as independent variables. The results show that larger non-family networks were linked with increased social participation in both societies. Men were more vulnerable to engaging in no social activities and at a higher risk of social isolation in both countries. One difference between the two nations is that among the Japanese, people with higher social orientations engage in more *active social* type participation.

**Conclusion:**

This study reveals that non-kin social networks are important for social participation in Japan and Korea.

## Introduction

### Social background

#### Population aging in Japan and Korea

One of the biggest challenges developed countries face today is population aging. The proportion of elderly citizens aged 65 and older constitutes 26.7% of the total population in Japan [1]. Korea’s proportion of aging citizens was 13.1% in 2015, which is not as high, but it is aging more rapidly than any other country and is expected to see higher aging rates than Japan by the 2040s.

In both countries, birth rates are decreasing as their elderly populations are increasing. The 2015 total fertility rate, which shows the average number of children a woman births in a lifetime, was 1.45 in Japan and 1.25 in Korea. These low figures will result in decreased working populations to support the countries’ economies. One of the strategies to address this situation is for those over 65 years old, who were thought to be a dependent population, to continue working as long as possible. In Japan, the Elderly Persons Employment Stabilization Act was amended in 2013, which enables continued employment up to 65 years of age.

**Fig 1:**
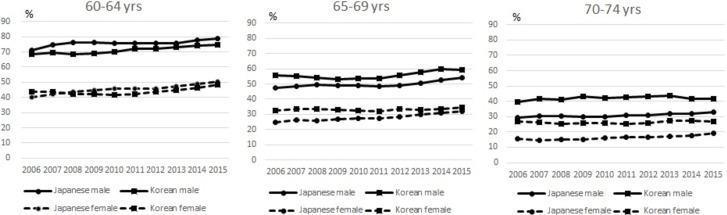
Changes in working population rates in Japan and Korea. Source: Organization for Economic Co-operation and Development (OECD) Stat (9 February 2017). http://stats.oecd.org/.

Fig 1 shows the changing rates in the workforce among 60 to 74-year-olds from 2006 to 2015 by country and gender.

Although working rates among the elderly have increased, a retirement age is still maintained by 73.5% of Japanese companies, of which 74.9% set this age at 60 [2]. Most employment contracts end at 60 and a new short-term contract is signed, which usually contains worse conditions for older employees than when they were younger. This poses a challenge for the elderly to maintain their morale and motivation to continue working as they did in their prime years.

In Korea, people leave their employment in their late 40s to 50s under the name of voluntary retirement. This life stage coincides with home loan payments and paying school tuition for children, but people are often forced to take on a second job and work under poor conditions.

#### The need for social participation

The most important aspects of social participation are its positive effects on subjective well-being [3, 4] and the fact that it provides people with meaning in their lives. In a study of the effects of social participation on men in their 60s, social participation had positive effects on self-esteem for all men; however, when interactions between social participation and employment were considered, social participation had a greater effect on self-esteem when the respondents were employed than when they were retired. This finding indicates that after 60, work is no longer a source of one’s purpose for life and social participation compensates for this loss [5].

Second, social participation is expected to prevent social isolation. Social isolation is a major risk factor for morbidity and mortality [6]. In both Japan and Korea, those who were long-term employees of a company had few acquaintances or connections with their community in their prime years. If employees do not participate in their local communities, they will be at risk of isolation when they retire. Additionally, the family composition is changing. Both Japanese and Korean families formerly consisted of extended, patriarchal families, but modernization and urbanization have created nuclear ones instead. An international survey shows that the ratio of people living alone rose from 5.7% in 1980 to 12.8% in 2010 in Japan, and from 4.3% to 21.0% in Korea [7]. Although these percentages are low compared to developed, Western countries, this is a new trend for Japan and Korea. Studies indicate that single elderly households have higher risks of social isolation. The ratio of people who do not have contact with non-relatives is 26.2% in Japan and 31.3% in Korea; these figures are higher than in Western countries [7].

One way to prevent social isolation is through social participation [3]. Having finished raising children and retiring from various social roles, the elderly have more free time, and social participation forms a major part of their lives. Social participation not only prevents isolation, but the effective use of free time and active social participation are the most important factors that determine satisfaction in the elderly life stage [8].

Social participation among the Japanese elderly (60 and older) increased from approximately 40% to 60% in the past 20 years due to the Japanese government’s initiative to promote participation. However, participation in urban areas is less than 40% and decreasing, resulting in a widening gap between regions [3].

A survey by the Korean Statistics Institute on the participation of the elderly (65 and older) also shows increased participation rates. However, the 2015 ratio is 40%, which is lower than in Japan.

### Theoretical background

#### Various forms of social participation

The literature describes various definitions for social participation, but there seems to be no consensus on a theoretical definition [3, 9]. Some studies [10] address social participation as volunteer activities, while others [11] consider socializing with other people to be social participation. Sometimes, when the elderly do housework, they are regarded as engaging in a social activity compared to others taking care of them on a daily basis [12, 13]. Although social participation includes various forms of activities, social interactions and productive activities have been the primary research focus in social participation literature.

Few studies have attempted to categorize social participation with a particular focus. Bukov et al. [14] categorized participation as either collective social participation or productive political social participation according based on shared resources, while Levasseur et al. [15] classified social participation based on six levels of interactions with others. Katagiri [3] suggests breaking down social participation into four phases depending on the orientation of the activity and the level of successful aging. It is necessary to categorize social participation according to the type of activity involved, but only a few studies have suggested such categorization. It is necessary to consider how distinct kinds of activities affect social participation, since the effects of each type could differ.

Factors relating to participation and their effects vary according to the level of participation, as well as the type of activity. Minagawa and Saito [16] claimed that it was necessary to consider the level of participation, although they only used group membership in their analysis. Only Parker [17] proposed classifying social activities by the extent of a person’s involvement. Most studies have not taken into account the level of participation, and used the simple categorization of *participating* or *not participating*. It is critical to consider the various levels of involvement in social participation since they probably relate to different factors. At the same time, different levels of social participation could have distinct effects on seniors.

#### Factors related to social participation

Katagiri [3] reviewed studies on social participation in Japan and derived factors that promote and prevent social participation according to the following criteria: a) basic attributes (the presence of one’s spouse, lifestyle, education, health conditions, etc.); b) personal resources (amount of free time, activities of daily living, active-life orientation, affiliation motivation, and attachment to community); c) social resources (number of close friends and neighbors, number of those expected to provide social support, etc.); and d) macro-social factors (newly built towns, unsatisfactory government services, few groups designed for retired men, most groups dominated by women, etc.). The present review notes that common factors are not found in past studies because they focused on different types of social participation, people with distinct attributes, and different regions.

More recent studies show that other factors come into play, such as elements related to personal history, the number of years the respondent has lived in the area, whether the respondent lived in the area before retirement, and past experience with social participation [18–20]. Employment might inhibit social participation [21]. Okamoto showed that social resources, such as one’s number of friends and acquaintances, also influence social participation [19, 20].

In Korea, numerous studies have been done on the factors related to community activity and social participation, and found outcomes similar to the results of Japanese investigations.

One of the drawbacks of research in both countries is that only a few have examined the abovementioned issues by nationally representative sample. In addition, some investigations have suggested that rural and urban areas have differences in social participation [5, 22]; however, little research has been carried out on this point.

### Aims

As discussed above, the social participation of the elderly is an important issue both in Japan and Korea. Examining the factors affecting the social participation of the elderly is an urgent matter. However, although many studies have been conducted on volunteering and the associated determining factors in Western countries, systematic research on social participation as a whole has not been conducted [3]. Moreover, due to social and cultural differences, some findings in Western countries might not apply to Japan and Korea.

Japan and Korea share similar values such as Confucianism; thus, a comparison of the two countries holds significance. As discussed above, the level of social participation in both countries is not yet adequate, and given their similar backgrounds (compared to Western countries), examining the determining factors of social participation is not only socially meaningful, but theoretically significant as well. Past literature on social participation in both countries has used nationally representative data, which are limited; more investigations using nationally representative data should be carried out. This study aims to first examine and compare the factors relating to social participation in Japan and Korea. Second, the types of activities will be categorized and related factors will be compared among different types of social participation. Third, most studies use a dichotomous classification of *participation* and *no participation*. This study designates the level of participation as *no affiliation* (respondent does not belong to any group), *inactive participation* (respondent belongs to a group but does not engage in activities), *active recreational participation* (respondent is actively involved in a recreational group) and *active social participation* (respondent is actively involved in a social group). This study explores how related factors differ among the four groups. Fourth, this study analyzes nationally representative samples and looks at the differences between urban and rural areas.

## Methods

### Data

A secondary analysis was performed using Japanese and Korean data from the 2012 East Asia Social Survey (EASS), which asked respondents the same questions in Japan, Korea, China, and Taiwan.

The Japanese data included a national sample of 4,500 males and females aged 20 to 89 living in Japan, selected by two-stage stratified random sampling and stratified by regional block and population size. Data were collected through both face-to face interviews and placement methods. The response rate was 58.8%.

The Korean data included a national sample of 2,500 males and females 18 and older selected by multi-stage area probability sampling. All questions were asked through face-to-face interviews. The response rate was 55.8%.

Only data from respondents 65 and older were included in the analyses (Japanese: *N* = 683, Koreans: *N* = 362). For details on the survey methods, please see the 2014 article by the Japanese General Social Surveys (JGSS) [23].

### Items included in the survey

#### Dependent variables

EASS 2012 asks respondents about their participation in seven types of activities: political associations, residential/neighborhood associations, social service clubs (volunteer groups/non-profit organizations or NPOs), citizen movement/consumer cooperative groups, religious groups, alumni associations, and recreational associations (hobbies and sports). Labor unions and occupational/professional associations/trade associations were included, but not in the present analyses since this study focuses on social participation other than work. For each item, respondents were asked to choose from the following responses: *yes*, *actively involved*; *yes*, *but hardly involved*; or *no*.

First, the seven activities were categorized by purpose into two types. Alumni associations and recreational associations (hobbies and sports) were classified as *recreational*; religious groups, political associations, residential/neighborhood associations, social service clubs (volunteer groups and NPOs), and citizen movement/consumer cooperative groups were designated as *social*. Participation in religious groups was also considered *social*, although religious activities are not always social, but rather very personal pursuits such as engaging in silent prayer; they cannot be regarded as recreational. Furthermore, religious groups were often seen doing volunteer work, so they were combined with social activities. In addition, many Koreans engage in religious activity but only few Japanese do. This was not sufficient to create a separate category for religious activity. Thus, two categories were employed.

Next, the degree of active engagement in each activity was considered. The responses include *yes*, *actively involved*; *yes*, *but hardly involved*; or *no*. We measured the degree of active engagement according to this answer. For example, when the respondent chose *yes*, *actively involved* for alumni associations or recreational associations (hobbies and sports), the respondent was categorized as *active recreational*. Those people categorized as both *active recreational* and *active social* were categorized into *active social*. Since the models of a hierarchical level of social participation assumed that activities included in ‘*active social*’ in this study are of a higher level than those in ‘*active recreational*’ [3,14,15].

Finally, the following four categories were created: *no affiliation* where people did not join any of the groups; *inactive participation* where people were affiliated with at least one group but not actively participating; *active recreational*, whereby respondents actively took part in recreational activities; and *active social*, for which respondents actively engaged in other activities. As a result, a categorical variable for social participation was created with 0 for *no affiliation*, 1 for *inactive participation*, 2 for *active recreational*, and 3 for *active social*. These categories include both the type of participation and level of involvement.

#### Independent variables

The factors relating to social participation in Japan and Korea, as suggested in the prior research, were considered independent variables as classified by Katagiri [3]. These were *basic attributes*, *personal resources*, *social resources*, and *macro-social* factors.

The variables for basic attributes were as follows: dummy variables for age with *young-old* (65 to 74) as 0 and *old-old* (older than 75) as 1; a five-point scale of subjective health from 1, *very bad* to 5, *very good*; a five-point scale of household income from 1, *far below average* to 5, *far above average*; a categorical variable on *living alone* (1) or *not living alone* (0); a categorical variable on educational level, with (1) designating *junior high school or less*, (2) representing *high school*, and (3) indicating *beyond high school*; *being employed* (1) *or not employed* (0); years living in the community with 1 as *less than a year*, 2 as *1–2*.*99 years*, 3 as *3–4*.*99 years*, 4 as *5–9*.*99 years*, 5 as *10–19*.*99 years*, 6 as *20–29*.*99 years*, 7 as *30 years and above*, and 8 as *since I was born*.

Personal resources included the *social contribution orientation*, which was measured by how much the person wanted to contribute to society on a 7-point scale with 1 as *strongly disagree* and 7 as *strongly agree*.

Regarding social resources, “the number of family members and relatives one has contact with in a day” was measured on an eight-point scale using the question, “On an ordinary day, with how many family members or relatives–excluding those who live with you–do you have contact through telephone, mail, the Internet, or face-to-face?” Respondents chose 1 for *0*, 2 for *1–2*, 3 for *3–4*, 4 for *5–9*, 5 for *10–19*, 6 for *20–49*, 7 for *50–99*, or 8 for *100 or more*. “The number of people who are not family members that one has contact with in a day” was measured using the question, “On an ordinary day, with how many people other than family members or relatives do you have contact through telephone, mail, the Internet, or face-to-face?” Respondents used the same eight-point scale that was employed for the question on contact with family and relatives. *Neighborhood network* was measured using the question, “With how many neighbors are you on greeting terms?” Respondents chose from a five-point scale with1 for *0*, 2 for *1–2*, 3 for *3–4*, 4 for *5–9*, 5 for *10 or more*.

For *macro-social* factors, the level of urbanization was measured on a five-point scale with 1 as *a farm or home in the country* to 5 as *a big city* according to the respondent’s evaluation.

#### Analyses

Data were separated into four groups by country and gender, and multinomial logistic regression analyses were performed for each group, with *social participation* as the dependent variable (*inactive participation* was the reference group); the independent variables described above were used as explanatory variables. The analyses excluded respondents with missing data from any variable in the model; thus, complete case analyses were performed assuming missingness completely at random (MCAR). We also performed the analyses using imputation by EM estimation (S1 Table), and multiple linear regression (S2 Table), assuming missing at random (MAR) for sensitivity analysis. We then compared the results with the complete case analyses, and with the multiple imputation analyses.

The software SPSS Version 24 was used for the analyses.

## Results

### Descriptive statistics

Table 1 displays the breakdown of basic attributes and independent variables for both the Japanese and Korean respondents. The age groups were similar in both countries. The percentage of women was slightly higher in Korea (58.8%) than in Japan (54.8%); more Koreans (30.9%) were working than Japanese (24.3%); more than double the number of Koreans (39.5%) lived alone, versus their Japanese counterparts (16.7%); and approximately 20% more Japanese (72.2%) were married than Koreans (54.1%). The Japanese respondents had higher levels of education: 19.5% of Japanese interviewees had an education beyond high school, while that figure was 9.4% for Koreans; 45.1% of Japanese and 13.6% of Koreans finished high school; and 35.5% of Japanese and 76.9% of Koreans completed junior high school or less.

**Table 1:** Descriptive statistics.

	Japanese (N = 683)		Koreans (N = 362)	
	Mean	*SD*	Mean	*SD*
Age	65.8	9.32	65.3	10.3
Subjective health (1: Very bad—5: Very good)	3.4	0.98	2.9	1.2
Household income (1: Far below average—5: Far above average)	2.5	0.91	2.3	1.0
Years living in the community (1: Less than a year, 2: 1–2.99 years, 3: 3–4.99 years, 4: 5–9.99 years, 5: 10–19.99 years, 6: 20–29.999 years, 7: 30 years and more, 8: since I was born)	6.2	1.46	5.8	1.7
Number of people one contacts daily (family/relatives) (1: 0, 2: 1–2, 3: 3–4, 4: 5–9, 5: 10–19, 6: 20–49, 7: 50–99, 8: 100 or more)	2.4	1.19	2.3	0.9
Number of people one contacts daily (other than family/relative) (1: 0, 2: 1–2, 3: 3–4, 4: 5–9, 5: 10–19, 6: 20–49, 7: 50–99, 8: 100 or more)	3.6	1.63	3.0	1.3
With how many neighbors are you on greeting terms? (1: 0, 2: 1–2, 3: 3–4, 4: 5–9, 5: 10 or more)	3.6	1.12	4.1	1.2
Wish to make contributions towards society (1:strongly disagree—7: strongly agree)	4.8	1.06	4.5	1.6
Level of urbanization (1: a farm or home in the country—5: a big city)	2.8	0.90	3.1	1.2

Values for subjective health, subjective financial well-being, and years living in the community were all significantly higher for the Japanese. Furthermore, social contribution orientation was higher in Japan.

Regarding social resources, there were no differences in the number of family members or relatives one has contact with in a day, and values for neighborhood networks and non-family contacts were higher in Japan. There was no significant difference in community urbanization.

### Social participation

Table 2 shows social participation for the seven types of groups and levels of participation for respondents in each nation. In both countries, local community groups had the highest level of participation. Approximately 71% of Japanese said they participated in local community groups but 65% of them said they were not actively involved. In Korea, 41% participated, of which 40% were inactively involved. On the whole, approximately 25% of respondents actively participated in local communities in both countries, revealing little difference.

**Table 2:** Social participation (group category) and active involvement by country.

		No	Yes, but hardly involved	Yes, actively involved
Political participation	Japanese	93%	5%	2%
	Koreans	96%	3%	1%
Residential/neighborhood groups	Japanese	30%	46%	25%
	Koreans	59%	17%	25%
Social service clubs	Japanese	89%	6%	5%
	Koreans	88%	4%	8%
Citizen movement	Japanese	85%	12%	4%
	Koreans	96%	2%	2%
Religious groups	Japanese	87%	10%	4%
	Koreans	70%	13%	17%
Alumni groups	Japanese	49%	28%	23%
	Koreans	72%	13%	14%
Recreational groups	Japanese	63%	9%	28%
	Koreans	77%	7%	15%

The category with the second highest participation level (although not among those actively involved) was alumni associations. Affiliations included 51% of Japanese and 28% of Koreans; of these total amounts, 55% of Japanese and 48% Koreans were inactive.

After local community groups and alumni associations, Japanese participation was high for hobbies and sports groups. In Korea, the order was local community groups, then religious groups, followed by alumni associations.

Table 3 shows four types of participation in each group by country and gender, showing percentages for *no affiliation*, *inactive*, *active recreational*, and *active social*. More Koreans (30%) were in *no affiliation* than Japanese (15%), whereas 37% Japanese were *inactive*, and only 19% of Koreans were *inactive*. This suggests that the Japanese tended to be nominally affiliated with groups. Respondents who were *active recreational* and *active social* amounted to 21% in Japan and 10% in Korea, and 27% and 41%, respectively. Slightly more men (33%) than women (29%) were *inactive*. The category of *active recreational* was found to be 18% for men and 16% for women, while 33% of men and 31% of women were *active social*.

**Table 3:** Social participation type.

		No affiliation	Inactive	Active recreational	Active social
Japanese	Male	35 (11%)	119 (39%)	62 (20%)	93 (30%)
	Female	67 (18%)	134 (36%)	79 (21%)	94 (25%)
Koreans	Male	37 (25%)	30 (20%)	22 (15%)	60 (40%)
	Female	73 (34%)	38 (18%)	13 (6%)	89 (42%)

### Factors relating to social participation

#### Results for the Japanese

Multinomial logistic regression analyses were performed separately for each country. The *inactive participation* type was the reference group. In Japan, compared to *inactive*, *no affiliation* included men who lived alone, graduated from more than high school were less likely in *no affiliation* compared to people graduated from junior high school or less (Table 4). Across men and women, *active recreational* tended to be highest for the young-old age group and those with better subjective health. Among women, those who were not employed, with higher household incomes, and had more contact with non-relative members tended to participate in *recreational*-type groups.

**Table 4:** Multinomial logistic regression analysis for the Japanese sample.

	Japanese men (*N*=291)									Japanese women (*N*=333)								
	*b*	*SE*	*p*	Odds ratio		95% CI				*b*	*SE*	*p*	Odds ratio		95% CI			
**No affiliation type**																		
Intercept	.39	1.88								-.68	1.32							
Old-old (1: yes, 0: no)	.00	.48		1.00	[	.40	,	2.54	]	.15	.36		1.16	[	.57	,	2.35	]
Education (reference group = junior high school or less)																		
High school	.05	.47		1.05	[	.41	,	2.65	]	.24	.35		1.27	[	.64	,	2.53	]
More than high school	-1.82	.86	*	.16	[	.03	,	.87	]	-2.07	1.08		.13	[	.02	,	1.04	]
Subjective health (1: very bad - 5: very good)	.16	.22		1.18	[	.76	,	1.81	]	.00	.17		1.00	[	.71	,	1.40	]
Household income (1: far below average - 5: far above average)	-.12	.27		.89	[	.52	,	1.52	]	-.17	.22		.84	[	.55	,	1.30	]
Living alone (1: yes, 0: no)	1.41	.62	*	4.08	[	1.20	,	13.83	]	.09	.41		1.09	[	.49	,	2.43	]
Employed (1: yes, 0: no)	-.09	.53		.92	[	.32	,	2.61	]	-.65	.48		.52	[	.20	,	1.33	]
Years living in the community	-.09	.15		.92	[	.68	,	1.24	]	-.04	.12		.96	[	.76	,	1.23	]
Wish to make contributions towards society	.18	.21		1.20	[	.80	,	1.79	]	-.06	.15		.94	[	.70	,	1.27	]
Number of people one contacts daily (family/relatives)	.17	.24		1.18	[	.74	,	1.88	]	.08	.17		1.08	[	.77	,	1.53	]
Number of people one contacts daily (other than family/relatives)	-.37	.23		.69	[	.44	,	1.09	]	.19	.17		1.21	[	.87	,	1.68	]
With how many neighbors are you on greeting terms?	-.38	.21		.68	[	.45	,	1.04	]	-.03	.16		.97	[	.71	,	1.32	]
Level of urbanization (1: rural - 5: urban)	-.11	.24		.89	[	.56	,	1.43	]	.12	.18		1.12	[	.79	,	1.60	]
**Active recreational type**																		
Intercept	-3.99	1.55	**							-4.27	1.44	***						
Old-old (1: yes, 0: no)	-.81	.39	*	.45	[	.21	,	.95	]	-.87	.37	*	.42	[	.20	,	.87	]
Education (reference group = junior high school or less)																		
High school	.35	.44		1.42	[	.60	,	3.38	]	.67	.39		1.95	[	.92	,	4.15	]
More than high school	.87	.47		2.38	[	.94	,	6.00	]	.91	.51		2.49	[	.93	,	6.70	]
Subjective health (1: very bad - 5: very good)	.42	.18	*	1.52	[	1.07	,	2.17	]	.59	.17	***	1.80	[	1.29	,	2.49	]
Household income (1: far below average - 5: far above average)	.13	.20		1.14	[	.77	,	1.69	]	.45	.23	*	1.56	[	1.00	,	2.45	]
Living alone (1: yes, 0: no)	.92	.57		2.50	[	.82	,	7.65	]	.52	.42		1.68	[	.74	,	3.80	]
Employed (1: yes, 0: no)	-.43	.42		.65	[	.29	,	1.47	]	-1.87	.52	***	.16	[	.06	,	.43	]
Years living in the community	.11	.13		1.11	[	.86	,	1.45	]	-.02	.12		.98	[	.77	,	1.25	]
Wish to make contributions towards society	.14	.18		1.16	[	.81	,	1.65	]	.07	.15		1.07	[	.80	,	1.45	]
Number of people one contacts daily (family/relatives)	-.01	.16		1.00	[	.73	,	1.35	]	-.17	.18		.85	[	.59	,	1.20	]
Number of people one contacts daily (other than family/relatives)	.06	.15		1.06	[	.79	,	1.43	]	.36	.17	*	1.44	[	1.02	,	2.02	]
With how many neighbors are you on greeting terms?	.04	.16		1.04	[	.75	,	1.43	]	.02	.16		1.02	[	.75	,	1.39	]
Level of urbanization (1: rural - 5: urban)	-.05	.19		.96	[	.65	,	1.40	]	-.08	.19		.92	[	.64	,	1.33	]
**Active social type**																		
Intercept	-7.32	1.62	***							-5.80	1.45	***						
Old-old (1: yes, 0: no)	-.75	.35	*	.47	[	.24	,	.94	]	-.42	.33		.66	[	.34	,	1.25	]
Education (reference group = junior high school or less)																		
High school	-.10	.38		.91	[	.44	,	1.90	]	.80	.35	*	2.22	[	1.12	,	4.41	]
More than high school	-.14	.45		.87	[	.36	,	2.10	]	.81	.49		2.24	[	.86	,	5.80	]
Subjective health (1: very bad - 5: very good)	.26	.17		1.30	[	.94	,	1.79	]	.28	.16		1.33	[	.98	,	1.80	]
Household income (1: far below average - 5: far above average)	.22	.19		1.25	[	.86	,	1.83	]	.02	.21		1.02	[	.68	,	1.53	]
Living alone (1: yes, 0: no)	.79	.59		2.21	[	.69	,	7.04	]	-.09	.41		.91	[	.41	,	2.02	]
Employed (1: yes, 0: no)	-.48	.38		.62	[	.30	,	1.31	]	-.62	.39		.54	[	.25	,	1.14	]
Years living in the community	.40	.16	**	1.49	[	1.10	,	2.02	]	.17	.13		1.19	[	.92	,	1.54	]
Wish to make contributions towards society	.45	.17	**	1.57	[	1.13	,	2.18	]	.29	.15	*	1.34	[	1.00	,	1.79	]
Number of people one contacts daily (family/relatives)	.12	.14		1.13	[	.85	,	1.50	]	.33	.16	*	1.39	[	1.02	,	1.89	]
Number of people one contacts daily (other than family/relatives)	.08	.14		1.09	[	.83	,	1.42	]	.06	.16		1.06	[	.77	,	1.46	]
With how many neighbors are you on greeting terms?	.42	.16	**	1.51	[	1.11	,	2.06	]	.14	.15		1.15	[	.86	,	1.55	]
Level of urbanization (1: rural - 5: urban)	-.33	.18		.72	[	.50	,	1.02	]	.08	.16		1.09	[	.79	,	1.49	]
-2log likelihood	645.98									799.74								
*df*	39									39								
Pseudo-R^2^ Nagelkerke	.34									.28								

Reference category is 'inactive type'.

Note.

* p < .05

** p < .01

*** p < .001.

Across men and women, those with a higher social contribution orientation were more likely to participate as *social* compared to those who were *inactive*. Men who were in the young-old category, had lived longer in the community, and had larger neighborhood networks tended to participate in *social*-type groups, while women who had graduated from high school (compared to junior high school), and had more contact with family and relatives tended to participate in *social*-type groups.

#### Results for Koreans

Compared to *inactive*, more men tended to be in *no affiliation* when they had lower subjective health, smaller neighborhood networks, and lived in a more rural area (Table 5).

**Table 5:** Multinomial logistic regression analysis for the Korean sample.

	Korean men (*N* = 144)									Korean women (*N* = 196)								
	*b*	*SE*	*p*	Odds ratio		95% CI				*b*	*SE*	*p*	Odds ratio		95% CI			
**No affiliation type**																		
Intercept	4.73	2.48								1.75	1.78							
Old-old (1: yes, 0: no)	.98	.68		2.66	[	.70	,	10.17	]	.02	.45		1.02	[	.42	,	2.48	]
Education (reference group = junior high school or less)																		
High school	.31	.77		1.37	[	.31	,	6.13	]	-1.42	1.33		.24	[	.02	,	3.28	]
More than high school	.67	.97		1.96	[	.29	,	13.16	]	-1.44	1.58		.24	[	.01	,	5.26	]
Subjective health (1: very bad—5: very good)	-.61	.29	*	.54	[	.31	,	.96	]	-.25	.21		.78	[	.51	,	1.18	]
Household income (1: far below average—5: far above average)	.39	.32		1.48	[	.80	,	2.76	]	.53	.31		1.69	[	.92	,	3.11	]
Living alone (1: yes, 0: no)	.62	.77		1.86	[	.42	,	8.34	]	.51	.48		1.67	[	.65	,	4.30	]
Employed (1: yes, 0: no)	.01	.63		1.01	[	.29	,	3.43	]	-.56	.62		.57	[	.17	,	1.95	]
Years living in the community	-.12	.18		.89	[	.63	,	1.26	]	-.14	.18		.87	[	.61	,	1.23	]
Wish to make contributions towards society	.03	.20		1.03	[	.69	,	1.54	]	.05	.15		1.05	[	.79	,	1.41	]
Number of people one contacts daily (family/relatives)	.29	.32		1.34	[	.72	,	2.48	]	-.04	.29		.96	[	.55	,	1.69	]
Number of people one contacts daily (other than family/relatives)	.02	.31		1.02	[	.55	,	1.88	]	.08	.21		1.09	[	.73	,	1.63	]
With how many neighbors are you on greeting terms?	-.63	.26	**	.53	[	.32	,	.88	]	-.11	.21		.90	[	.59	,	1.36	]
Level of urbanization (1: rural—5: urban)	-.68	.31	*	.51	[	.28	,	.93	]	-.21	.22		.81	[	.53	,	1.24	]
**Active recreational type**																		
Intercept	-3.48	2.98								.49	2.77							
Old-old (1: yes, 0: no)	-.82	.82		.44	[	.09	,	2.20	]	-1.64	.91		.20	[	.03	,	1.16	]
Education (reference group = junior high school or less)																		
High school	.29	.82		1.34	[	.27	,	6.64	]	.55	1.22		1.74	[	.16	,	19.06	]
More than high school	1.81	.92	*	6.11	[	1.01	,	37.07	]	.63	1.57		1.89	[	.09	,	40.62	]
Subjective health (1: very bad—5: very good)	-.09	.30		.92	[	.52	,	1.64	]	-.27	.38		.76	[	.36	,	1.61	]
Household income (1: far below average—5: far above average)	.12	.35		1.13	[	.56	,	2.27	]	.69	.47		2.00	[	.80	,	5.00	]
Living alone (1: yes, 0: no)	-.31	1.00		.74	[	.10	,	5.22	]	-.57	.78		.57	[	.12	,	2.63	]
Employed (1: yes, 0: no)	.19	.67		1.21	[	.32	,	4.52	]	.46	.86		1.59	[	.30	,	8.49	]
Years living in the community	.19	.24		1.21	[	.76	,	1.92	]	-.35	.25		.70	[	.43	,	1.15	]
Wish to make contributions towards society	.02	.22		1.02	[	.66	,	1.56	]	.08	.25		1.08	[	.67	,	1.76	]
Number of people one contacts daily (family/relatives)	-.29	.34		.75	[	.39	,	1.45	]	.01	.46		1.01	[	.41	,	2.50	]
Number of people one contacts daily (other than family/relatives)	.65	.32	*	1.92	[	1.03	,	3.59	]	-.20	.37		.82	[	.40	,	1.70	]
With how many neighbors are you on greeting terms?	.02	.29		1.02	[	.58	,	1.82	]	.09	.35		1.09	[	.55	,	2.18	]
Level of urbanization (1: rural—5: urban)	.15	.32		1.17	[	.63	,	2.17	]	.08	.35		1.08	[	.54	,	2.16	]
**Active social type**																		
Intercept	-.67	2.39								-.56	1.82							
Old-old (1: yes, 0: no)	.19	.65		1.21	[	.34	,	4.34	]	-.66	.45	** **	.52	[	.21	,	1.24	]
Education (reference group = junior high school or less)																		
High school	-.27	.68		.76	[	.20	,	2.87	]	.51	.93		1.66	[	.27	,	10.28	]
More than high school	1.02	.86		2.77	[	.52	,	14.85	]	-1.09	1.40		.34	[	.02	,	5.22	]
Subjective health (1: very bad—5: very good)	.04	.25		1.04	[	.64	,	1.68	]	.02	.21		1.02	[	.68	,	1.53	]
Household income (1: far below average—5: far above average)	-.13	.30		.88	[	.49	,	1.57	]	.52	.30		1.68	[	.92	,	3.04	]
Living alone (1: yes, 0: no)	-.72	.87		.49	[	.09	,	2.65	]	.30	.47		1.35	[	.54	,	3.36	]
Employed (1: yes, 0: no)	.41	.56		1.51	[	.51	,	4.51	]	.39	.56		1.47	[	.49	,	4.41	]
Years living in the community	-.04	.18		.96	[	.67	,	1.38	]	-.09	.18		.92	[	.64	,	1.31	]
Wish to make contributions towards society	.17	.18		1.19	[	.83	,	1.70	]	.07	.15		1.07	[	.81	,	1.43	]
Number of people one contacts daily (family/relatives)	-.43	.30		.65	[	.36	,	1.17	]	.14	.27		1.15	[	.68	,	1.96	]
Number of people one contacts daily (other than family/relatives)	1.07	.28	***	2.91	[	1.67	,	5.05	]	.55	.21	**	1.74	[	1.16	,	2.61	]
With how many neighbors are you on greeting terms?	-.14	.24		.87	[	.55	,	1.38	]	-.19	.22		.83	[	.54	,	1.27	]
Level of urbanization (1: rural—5: urban)	-.21	.27		.81	[	.48	,	1.38	]	-.16	.21		.86	[	.56	,	1.30	]
-2log likelihood	301.61									406.96								
*df*	39									39								
Pseudo-R^2^ Nagelkerke	.45									.32								

Reference category is 'inactive type'.

Note.

* p < .05

** p < .01

*** p < .001.

Compared to the *inactive* type, more men took part in recreational groups when they had more contact with non-relatives and graduated from more than high school (compared to graduated from junior high school or less).

Across men and women, the larger the non-family network, the more respondents were inclined to participate in *social*-type groups compared to *inactive*-type groups.

Finally, we compared the results by complete case analyses (Table 4, Table 5) and by using imputation by EM estimation (S1 Table), and multiple linear regression (S2 Table), multiple imputations. Though there were some differences in the significance levels for different variables, we found substantially similar results.

## Discussion

### *No affiliation* and *inactive* participation

The rate of social participation was higher in Japan, but 37% of Japanese were *inactive*. This means they were affiliated with at least one group but were not actively involved, and the actual rate of participation was slightly higher in Korea. This finding indicates the need to closely investigate the state of participation, and suggests that national surveys might not be able to capture the actual state of involvement if they only ask respondents whether they participate in groups. This is an issue because social participation is thought to be effective only when it involves actual engagement.

In Japan, those who are not even nominally participating in any group tended to be men living alone. Since social participation and the associated social connections are thought to be important for psychological well-being after retirement, Japanese men are at risk of social isolation. In Korea, men with smaller neighborhood networks and who lived in rural areas tended to fall into the *no affiliation* type, which signals that men in rural parts of the country who have little contact with their neighbors are at higher risk of social isolation.

### *Active recreational* participation

In Japan, people who were younger and had better subjective health tended to participate in *recreational*-type groups. Additionally, women who were not employed were more actively involved in recreational groups due to having more free time. Having a larger network of non-relatives had a positive effect on recreational participation for Japanese women and Korean men.

### *Active social* participation

For *active social*, basic attributes were not related to participation in Koreans. Japanese younger men and Japanese women who had finished high school (compared to finished junior high school or less) were participating in this type.

Among Japanese men, those who had lived in the community for a long time and actively connected with neighbors were actively involved in *social*-type groups. This implies that retirees who moved to an area relatively recently and had few connections with neighbors had difficulty getting involved in *social*-type group activities compared to those who had lived in the area since childhood.

In Korea, only the size of non-relative networks affected *social*-type group participation.

Social contribution orientation had a positive relationship with *social*-type group participation in the Japanese sample, but not in the Korean one. Among the Japanese, strong community ties such as contact with neighbors, the number of years living in the community, and social contribution orientation explain *social*-type group participation, but this was not seen in Korea, where only network size had a positive relationship with *social*-type group participation. Rather, another factor might explain *social*-type group participation among the Korean elderly.

Network resources were examined as a factor relating to participation; as one becomes actively involved in *social*-type groups, the size of non-relative networks increases and the community network expands. One reason that networks were deemed a factor in relation to social participation is that they tend to facilitate the elderly’s involvement in *social*-type groups (the elderly tend to be introduced to groups by people in their networks). Thus, the larger the network, the greater the probability they will be introduced to a group, which can lead to active social participation. On the other hand, people who have little connection with the community during their working years and neighborhood networks might expand them after retirement due to active social participation. Katagiri [3] proposes a model whereby large networks facilitate social involvement, leading to an even bigger network. This study is a one wave, cross-sectional survey, and cannot examine cause and effect. Longitudinal research is thus necessary in the future.

### The significance of this research and challenges for the future

The most significant contribution of this research is the inclusion of the level of engagement and that it distinguishes between *inactive* participation and *no affiliation*. This study showed that complete non-affiliation leads to social isolation among both Japanese and Korean men.

Second, the study distinguished nominal from actual participation by examining related factors. Nominal participation greatly diminishes the merits of social participation; thus, it is important to examine the factors relating to actual active participation. In Japan, both personal and social resources are required for active participation, whereas in Korea, the key was a large network of non-relatives. Among Japanese men, who tend to be isolated from their communities, having local ties was an important factor for social participation. Additionally, social contribution orientation is an important factor of social participation.

Lastly, this study showed that comparisons between Japan and Korea are useful. Network size was important in both countries, which is in accordance with findings in Western countries. These factors can be considered universal elements for the promotion of social participation.

Regarding differences in the factors between the two countries, further research is needed to determine whether these arise from societal and cultural variations or differences in nationality. For example, the urbanization might differently influence on social participation. Careful consideration of the inhibiting or promoting factors in one country can provide valid suggestions for another.

This study also has some limitations that should be addressed. First, it used cross-sectional data. The causal relationship between the predictor variables of the model and the outcome variables could not be examined. It is necessary to analyze the model using longitudinal data. Second, although the original EASS 2012 included a national sample selected by two-stage stratified random sampling and stratified by regional block and population size for Japan, and comprised a national sample selected by multi-stage area probability sampling for Korea, the sample size used in this study was 683 for Japanese and 362 for Koreans. It is necessary to analyze the model with a larger sample size.

## Supporting information

S1 Table Multinomial logistic regression by EM imputation. (PDF)

S2 Table Multinomial logistic regression by imputation using multiple linear regression. (PDF)
