# Correction: Factors determining the social participation of older adults: A comparison between Japan and Korea using EASS 2012

**DOI:** 10.1371/journal.pone.0197865

**Published:** 2018-05-17

**Authors:** Keiko Katagiri, Ju-Hyun Kim

There are errors in the Funding section. The correct funding information is as follows: KK: This work was supported by JSPS KAKENHI Grant Number JP17H02626, Funder: Japan Society for the Promotion of Science, https://www.jsps.go.jp/english/. JHK & KK: This work was supported by National Research Foundation of Korea Grant Number NFR-2016-S1A3A2924582, Funder: National Research Foundation of Korea, https://www.nrf.re.kr/eng/main. The funders had no role in study design, data collection and analysis, decision to publish, or preparation of the manuscript.
