# Supplementary material for: Factors determining the social participation of older adults: A comparison between Japan and Korea using EASS 2012
Source: PLoS One. 2018 Apr 6;13(4):e0194703. doi: 10.1371/journal.pone.0194703 (PMC5889058; doi:10.1371/journal.pone.0194703)
Supplement: S1 Table — (PDF) [file pone.0194703.s001.pdf]

S1. Multinomial logistic regression by EM imputation

	Japanese men (N=309)						Japanese women (N=374)					
	b	SE	p	Odds ratio	95% CI		b	SE	p	Odds ratio	95% CI	
**No affiliation type**												
Intercept	.28	1.73					.13	1.22				
Old-old (1: yes, 0: no)	.03	.44		1.03	[.43	2.44]	.18	.33		1.19	[.62	2.29]
Education (reference group = junior high school or less)												
High school	.11	.45		1.12	[.46	2.69]	.09	.32		1.10	[.58	2.07]
More than high school	-1.27	.73		.28	[.07	1.18]	-2.20	1.08	*	.11	[.01	.92]
Subjective health (1: very bad - 5: very good)	.19	.21		1.21	[.80	1.83]	-.03	.16		.97	[.71	1.32]
Household income (1: far below average - 5: far above average)	-.12	.26		.89	[.53	1.47]	-.15	.20		.86	[.59	1.27]
Living alone (1: yes, 0: no)	1.13	.56	*	3.10	[1.04	9.28]	.03	.39		1.03	[.48	2.18]
Employed (1: yes, 0: no)	.00	.50		1.00	[.37	2.66]	-.26	.43		.77	[.33	1.78]
Years living in the community	-.06	.15		.94	[.71	1.25]	-.04	.11		.96	[.78	1.19]
Wish to make contributions towards society	.12	.20		1.13	[.77	1.66]	-.19	.14		.83	[.63	1.10]
Number of people one contacts daily (family/relatives)	.11	.22		1.12	[.72	1.74]	.03	.16		1.03	[.76	1.40]
Number of people one contacts daily (other than family/relatives)	-.35	.22		.70	[.46	1.09]	.14	.15		1.15	[.86	1.54]
With how many neighbors are you on greeting terms?	-.35	.20		.71	[.48	1.05]	-.05	.15		.96	[.71	1.28]
Level of urbanization (1: rural - 5: urban)	-.11	.23		.90	[.58	1.40]	.16	.17		1.17	[.85	1.62]
**Active recreational type**												
Intercept	-4.14	1.51	**				-4.16	1.35	***			
Old-old (1: yes, 0: no)	-.81	.37	*	.44	[.22	.92]	-.83	.35	*	.44	[.22	.86]
Education (reference group = junior high school or less)												
High school	.37	.42		1.45	[.63	3.33]	.51	.36		1.66	[.82	3.36]
More than high school	.96	.46	*	2.61	[1.06	6.41]	.90	.48		2.46	[.96	6.29]
Subjective health (1: very bad - 5: very good)	.41	.18	*	1.50	[1.07	2.12]	.54	.15	***	1.71	[1.26	2.31]
Household income (1: far below average - 5: far above average)	.13	.20		1.14	[.78	1.68]	.39	.21		1.48	[.98	2.23]
Living alone (1: yes, 0: no)	.65	.53		1.91	[.67	5.44]	.42	.40		1.51	[.70	3.30]
Employed (1: yes, 0: no)	-.50	.41		.61	[.28	1.34]	-1.72	.51	***	.18	[.07	.48]
Years living in the community	.14	.13		1.15	[.89	1.48]	-.03	.11		.97	[.78	1.21]
Wish to make contributions towards society	.10	.18		1.11	[.78	1.57]	.04	.15		1.05	[.79	1.39]
Number of people one contacts daily (family/relatives)	.00	.15		1.00	[.74	1.35]	-.15	.17		.86	[.62	1.19]
Number of people one contacts daily (other than family/relatives)	.06	.15		1.07	[.80	1.42]	.29	.16		1.34	[.98	1.84]
With how many neighbors are you on greeting terms?	.03	.16		1.03	[.75	1.41]	.06	.15		1.06	[.79	1.42]
Level of urbanization (1: rural - 5: urban)	.02	.19		1.02	[.71	1.47]	.07	.17		1.07	[.77	1.49]
**Active social type**												
Intercept	-7.11	1.55	***				-5.95	1.40	***			
Old-old (1: yes, 0: no)	-.71	.34	*	.49	[.25	.96]	-.56	.31		.57	[.31	1.06]
Education (reference group = junior high school or less)												
High school	.03	.36		1.03	[.51	2.08]	.75	.33	*	2.11	[1.10	4.05]
More than high school	-.01	.44		.99	[.42	2.34]	.89	.47		2.44	[.98	6.07]
Subjective health (1: very bad - 5: very good)	.29	.16		1.33	[.98	1.82]	.26	.15		1.30	[.98	1.73]
Household income (1: far below average - 5: far above average)	.24	.19		1.28	[.88	1.85]	.08	.20		1.08	[.74	1.59]
Living alone (1: yes, 0: no)	.54	.55		1.71	[.58	5.04]	-.01	.39		1.00	[.47	2.12]
Employed (1: yes, 0: no)	-.54	.37		.59	[.28	1.21]	-.35	.36		.70	[.35	1.43]
Years living in the community	.35	.15	*	1.41	[1.06	1.88]	.21	.13		1.24	[.96	1.59]
Wish to make contributions towards society	.44	.16	**	1.55	[1.12	2.12]	.25	.14		1.29	[.97	1.70]
Number of people one contacts daily (family/relatives)	.08	.14		1.08	[.82	1.43]	.28	.15		1.32	[.99	1.76]
Number of people one contacts daily (other than family/relatives)	.10	.13		1.10	[.85	1.43]	.05	.15		1.05	[.78	1.40]
With how many neighbors are you on greeting terms?	.41	.15	**	1.51	[1.12	2.05]	.10	.14		1.11	[.84	1.47]
Level of urbanization (1: rural - 5: urban)	-.32	.18		.73	[.51	1.03]	.16	.15		1.18	[.87	1.58]
-2log likelihood	694.8						899.2					
df	39						39					
Pseudo-R^2 Nagelkerke	.32						.28					

Reference category is 'inactive type'.

Note. * p < .05, ** p < .01, *** p < .001.

	Korean men (N = 149)						Korean women (N = 213)					
	b	SE	p	Odds ratio	95% CI		b	SE	p	Odds ratio	95% CI	
**No affiliation type**												
Intercept	3.96	2.34					2.39	1.77				
Old-old (1: yes, 0: no)	.89	.66		2.43	[.67	8.83]	-.25	.43		.78	[.34	1.82]
Education (reference group = junior high school or less)												
High school	.37	.73		1.45	[.35	6.03]	-2.27	1.21		.10	[.01	1.10]
More than high school	.51	.94		1.66	[.27	10.36]	-.53	1.44		.59	[.04	9.79]
Subjective health (1: very bad - 5: very good)	-.54	.27	*	.58	[.34	.99]	-.26	.20		.77	[.52	1.14]
Household income (1: far below average - 5: far above average)	.34	.31		1.41	[.77	2.60]	.56	.30		1.74	[.98	3.11]
Living alone (1: yes, 0: no)	.72	.75		2.06	[.48	8.90]	.40	.46		1.49	[.61	3.64]
Employed (1: yes, 0: no)	.07	.61		1.07	[.32	3.54]	-.73	.60		.48	[.15	1.56]
Years living in the community	-.18	.17		.84	[.60	1.18]	-.20	.18		.82	[.58	1.16]
Wish to make contributions towards society	.09	.20		1.09	[.74	1.61]	.04	.14		1.04	[.79	1.37]
Number of people one contacts daily (family/relatives)	.19	.30		1.21	[.67	2.20]	-.01	.27		.99	[.58	1.67]
Number of people one contacts daily (other than family/relatives)	-.02	.31		.99	[.54	1.79]	-.03	.19		.97	[.67	1.41]
With how many neighbors are you on greeting terms?	-.43	.23		.65	[.41	1.03]	-.04	.20		.96	[.64	1.42]
Level of urbanization (1: rural - 5: urban)	-.60	.30	*	.55	[.31	.98]	-.21	.21		.81	[.54	1.22]
**Active recreational type**												
Intercept	-3.84	2.96					.69	2.72				
Old-old (1: yes, 0: no)	-.92	.80		.40	[.08	1.91]	-1.72	.89	*	.18	[.03	1.03]
Education (reference group = junior high school or less)												
High school	.23	.81		1.26	[.26	6.10]	-.17	1.12		.85	[.09	7.65]
More than high school	1.74	.90		5.67	[.97	33.33]	.61	1.60		1.84	[.08	42.26]
Subjective health (1: very bad - 5: very good)	-.11	.29		.90	[.51	1.56]	-.26	.38		.77	[.37	1.64]
Household income (1: far below average - 5: far above average)	.07	.35		1.08	[.54	2.14]	.73	.47		2.07	[.83	5.20]
Living alone (1: yes, 0: no)	-.23	1.00		.79	[.11	5.64]	-.53	.77		.59	[.13	2.66]
Employed (1: yes, 0: no)	.08	.66		1.09	[.30	3.97]	.48	.85		1.61	[.31	8.46]
Years living in the community	.18	.24		1.19	[.75	1.90]	-.42	.25		.66	[.40	1.08]
Wish to make contributions towards society	.10	.21		1.10	[.72	1.67]	.09	.25		1.09	[.67	1.77]
Number of people one contacts daily (family/relatives)	-.33	.33		.72	[.38	1.38]	.08	.44		1.08	[.46	2.57]
Number of people one contacts daily (other than family/relatives)	.65	.31	*	1.91	[1.03	3.52]	-.32	.37		.73	[.35	1.49]
With how many neighbors are you on greeting terms?	.15	.28		1.16	[.67	2.02]	.14	.35		1.15	[.58	2.29]
Level of urbanization (1: rural - 5: urban)	.15	.31		1.16	[.63	2.14]	.09	.34		1.10	[.56	2.13]
**Active social type**												
Intercept	-1.58	2.34					.11	1.78				
Old-old (1: yes, 0: no)	.45	.63		1.57	[.46	5.37]	-.83	.42	*	.44	[.19	.99]
Education (reference group = junior high school or less)												
High school	-.38	.67		.68	[.19	2.51]	-.37	.76		.69	[.16	3.10]
More than high school	.94	.83		2.57	[.50	13.12]	-.97	1.43		.38	[.02	6.20]
Subjective health (1: very bad - 5: very good)	.08	.24		1.08	[.68	1.72]	-.01	.20		.99	[.68	1.45]
Household income (1: far below average - 5: far above average)	-.07	.29		.94	[.53	1.65]	.52	.29		1.69	[.96	2.97]
Living alone (1: yes, 0: no)	-.32	.82		.73	[.15	3.61]	.27	.44		1.31	[.56	3.07]
Employed (1: yes, 0: no)	.46	.55		1.58	[.54	4.63]	.28	.53		1.32	[.47	3.71]
Years living in the community	-.08	.18		.92	[.65	1.31]	-.11	.18		.89	[.63	1.27]
Wish to make contributions towards society	.23	.18		1.25	[.88	1.79]	.07	.14		1.07	[.82	1.40]
Number of people one contacts daily (family/relatives)	-.41	.29		.66	[.38	1.16]	.19	.25		1.21	[.74	1.97]
Number of people one contacts daily (other than family/relatives)	1.02	.27	***	2.77	[1.62	4.74]	.38	.19	*	1.46	[1.01	2.10]
With how many neighbors are you on greeting terms?	-.04	.22		.96	[.62	1.49]	-.18	.20		.84	[.56	1.25]
Level of urbanization (1: rural - 5: urban)	-.14	.26		.87	[.52	1.46]	-.13	.20		.88	[.59	1.31]
-2log likelihood	317.6						449.0					
df	39						39					
Pseudo-R^2 Nagelkerke	.43						.29					

Reference category is 'inactive type'.

Note. * p < .05, ** p < .01, *** p < .001.
