# Supplementary material for: Factors determining the social participation of older adults: A comparison between Japan and Korea using EASS 2012
Source: PLoS One. 2018 Apr 6;13(4):e0194703. doi: 10.1371/journal.pone.0194703 (PMC5889058; doi:10.1371/journal.pone.0194703)
Supplement: S2 Table — (PDF) [file pone.0194703.s002.pdf]

S2. Multinomial logistic regression by imputation using multiple linear regress

	Japanese men (*N*=309)						Japanese women (*N*=374)					
	b	*SE*	*p*	Odds ratio	95% CI		*b*	*SE*	*p*	Odds ratio	95% CI	
**No affiliation type**												
Intercept	.51	1.70					-.89	1.18				
Old-old (1: yes, 0: no)	.03	.44		1.03	[.43	2.45]	.22	.33		1.25	[.65	2.39]
Education (reference group = junior high school or less)												
High school	.09	.45		1.09	[.45	2.64]	.02	.32		1.02	[.54	1.91]
More than high school	-1.25	.73		.29	[.07	1.20]	-2.27	1.09	*	.10	[.01	.88]
Subjective health (1: very bad - 5: very good)	.18	.21		1.20	[.79	1.82]	-.07	.16		.93	[.68	1.27]
Household income (1: far below average - 5: far above average)	-.04	.26		.96	[.58	1.60]	-.26	.18		.77	[.54	1.10]
Living alone (1: yes, 0: no)	1.20	.57	*	3.33	[1.10	10.07]	-.03	.38		.97	[.46	2.06]
Employed (1: yes, 0: no)	-.02	.49		.98	[.37	2.56]	-.29	.43		.75	[.33	1.74]
Years living in the community	-.06	.15		.94	[.70	1.26]	-.03	.11		.97	[.78	1.20]
Wish to make contributions towards society	.04	.19		1.04	[.72	1.51]	.08	.10		1.09	[.89	1.33]
Number of people one contacts daily (family/relatives)	.14	.21		1.15	[.75	1.74]	.11	.15		1.12	[.83	1.51]
Number of people one contacts daily (other than family/relatives)	-.37	.21		.69	[.46	1.04]	.09	.15		1.09	[.81	1.46]
With how many neighbors are you on greeting terms?	-.35	.20		.71	[.48	1.04]	-.07	.15		.94	[.70	1.25]
Level of urbanization (1: rural - 5: urban)	-.12	.22		.89	[.57	1.38]	.23	.16		1.26	[.92	1.73]
**Active recreational type**												
Intercept	-4.02	1.46	*				-4.07	1.30	***			
Old-old (1: yes, 0: no)	-.82	.37	*	.44	[.21	.91]	-.82	.34	*	.44	[.22	.86]
Education (reference group = junior high school or less)												
High school	.36	.42		1.43	[.62	3.30]	.55	.36		1.73	[.86	3.49]
More than high school	.96	.46	*	2.60	[1.06	6.37]	.95	.47	*	2.57	[1.02	6.52]
Subjective health (1: very bad - 5: very good)	.40	.18	*	1.49	[1.06	2.10]	.54	.15	***	1.72	[1.27	2.32]
Household income (1: far below average - 5: far above average)	.16	.20		1.17	[.80	1.72]	.34	.20		1.41	[.96	2.08]
Living alone (1: yes, 0: no)	.69	.54		1.99	[.70	5.68]	.41	.40		1.50	[.69	3.26]
Employed (1: yes, 0: no)	-.49	.40		.61	[.28	1.34]	-1.71	.50	***	.18	[.07	.49]
Years living in the community	.13	.13		1.14	[.88	1.47]	-.02	.11		.98	[.79	1.22]
Wish to make contributions towards society	.08	.17		1.08	[.78	1.50]	.03	.13		1.03	[.80	1.32]
Number of people one contacts daily (family/relatives)	.04	.15		1.04	[.78	1.39]	-.12	.16		.89	[.65	1.23]
Number of people one contacts daily (other than family/relatives)	.04	.14		1.04	[.79	1.38]	.24	.16		1.27	[.94	1.73]
With how many neighbors are you on greeting terms?	.02	.16		1.02	[.74	1.40]	.08	.15		1.08	[.81	1.45]
Level of urbanization (1: rural - 5: urban)	.02	.19		1.02	[.71	1.47]	.06	.17		1.06	[.76	1.46]
**Active social type**												
Intercept	-6.33	1.48	***				-5.87	1.31	***			
Old-old (1: yes, 0: no)	-.70	.33	*	.50	[.26	.96]	-.56	.31		.57	[.31	1.05]
Education (reference group = junior high school or less)												
High school	.03	.36		1.03	[.51	2.08]	.83	.33	**	2.28	[1.20	4.36]
More than high school	.07	.43		1.07	[.46	2.51]	.95	.46	*	2.57	[1.04	6.37]
Subjective health (1: very bad - 5: very good)	.29	.16		1.34	[.98	1.82]	.28	.15	*	1.33	[1.00	1.76]
Household income (1: far below average - 5: far above average)	.26	.19		1.30	[.90	1.87]	.11	.18		1.11	[.78	1.59]
Living alone (1: yes, 0: no)	.60	.55		1.81	[.62	5.34]	.06	.38		1.06	[.50	2.25]
Employed (1: yes, 0: no)	-.54	.36		.58	[.29	1.19]	-.47	.37		.63	[.31	1.29]
Years living in the community	.33	.14	*	1.39	[1.05	1.84]	.26	.12	*	1.29	[1.01	1.64]
Wish to make contributions towards society	.27	.15		1.31	[.98	1.75]	.09	.10		1.10	[.90	1.33]
Number of people one contacts daily (family/relatives)	.13	.13		1.14	[.88	1.48]	.33	.14	*	1.39	[1.06	1.84]
Number of people one contacts daily (other than family/relatives)	.08	.13		1.08	[.84	1.39]	.00	.15		1.00	[.75	1.33]
With how many neighbors are you on greeting terms?	.42	.15	**	1.52	[1.12	2.05]	.15	.14		1.16	[.88	1.54]
Level of urbanization (1: rural - 5: urban)	-.31	.18		.74	[.52	1.04]	.18	.15		1.19	[.89	1.60]
-2log likelihood	698.6						899.6					
df	39						39					
Pseudo-R^2 Nagelkerke	.31						.28					

Reference category is 'inactive type'.

Note. * $p < .05$, ** $p < .01$, *** $p < .001$.

	Korean men (*N* = 149)						Korean women (*N* = 213)					
	b	*SE*	*p*	Odds ratio	95% CI		*b*	*SE*	*p*	Odds ratio	95% CI	
**No affiliation type**												
Intercept	4.01	2.30					2.41	1.77				
Old-old (1: yes, 0: no)	.89	.66		2.45	[.67	8.89]	-.25	.43		.78	[.34	1.82]
Education (reference group = junior high school or less)												
High school	.36	.73		1.43	[.35	5.91]	-2.20	1.21		.11	[.01	1.19]
More than high school	.51	.93		1.66	[.27	10.31]	-.87	1.35		.42	[.03	5.94]
Subjective health (1: very bad - 5: very good)	-.53	.27	*	.59	[.35	1.01]	-.26	.20		.77	[.52	1.14]
Household income (1: far below average - 5: far above average)	.26	.30		1.30	[.73	2.34]	.58	.29	*	1.79	[1.01	3.17]
Living alone (1: yes, 0: no)	.71	.75		2.03	[.47	8.78]	.38	.46		1.47	[.60	3.58]
Employed (1: yes, 0: no)	.02	.61		1.02	[.31	3.36]	-.76	.60		.47	[.15	1.53]
Years living in the community	-.19	.17		.83	[.59	1.16]	-.20	.18		.82	[.57	1.16]
Wish to make contributions towards society	.07	.20		1.08	[.73	1.58]	.06	.14		1.06	[.80	1.39]
Number of people one contacts daily (family/relatives)	.16	.30		1.17	[.65	2.12]	-.05	.27		.95	[.56	1.61]
Number of people one contacts daily (other than family/relatives)	.10	.30		1.11	[.61	2.01]	.00	.19		1.00	[.69	1.46]
With how many neighbors are you on greeting terms?	-.41	.23		.66	[.42	1.04]	-.06	.20		.94	[.63	1.40]
Level of urbanization (1: rural - 5: urban)	-.59	.30	*	.55	[.31	.99]	-.22	.21		.80	[.53	1.20]
**Active recreational type**												
Intercept	-3.80	2.96					.75	2.69				
Old-old (1: yes, 0: no)	-.89	.80		.41	[.09	1.98]	-1.72	.89	*	.18	[.03	1.03]
Education (reference group = junior high school or less)												
High school	.23	.81		1.25	[.26	6.11]	-.14	1.13		.87	[.10	7.92]
More than high school	1.77	.91	*	5.89	[1.00	34.85]	.34	1.50		1.41	[.07	26.65]
Subjective health (1: very bad - 5: very good)	-.12	.29		.89	[.51	1.55]	-.26	.38		.77	[.36	1.63]
Household income (1: far below average - 5: far above average)	.05	.35		1.05	[.53	2.08]	.77	.46		2.15	[.88	5.27]
Living alone (1: yes, 0: no)	-.24	1.00		.79	[.11	5.60]	-.53	.77		.59	[.13	2.66]
Employed (1: yes, 0: no)	.07	.66		1.07	[.29	3.94]	.47	.84		1.61	[.31	8.37]
Years living in the community	.18	.24		1.19	[.75	1.89]	-.42	.25		.66	[.40	1.08]
Wish to make contributions towards society	.10	.22		1.10	[.72	1.68]	.09	.25		1.10	[.68	1.78]
Number of people one contacts daily (family/relatives)	-.35	.33		.71	[.37	1.35]	.05	.44		1.05	[.45	2.47]
Number of people one contacts daily (other than family/relatives)	.69	.32	*	2.00	[1.08	3.71]	-.33	.37		.72	[.35	1.48]
With how many neighbors are you on greeting terms?	.14	.28		1.15	[.66	2.00]	.13	.35		1.14	[.57	2.28]
Level of urbanization (1: rural - 5: urban)	.15	.31		1.16	[.63	2.14]	.09	.34		1.09	[.56	2.12]
**Active social type**												
Intercept	-1.52	2.33					-.03	1.78				
Old-old (1: yes, 0: no)	.48	.63		1.62	[.47	5.59]	-.81	.42	*	.44	[.19	1.01]
Education (reference group = junior high school or less)												
High school	-.38	.67		.69	[.19	2.54]	-.31	.77		.73	[.16	3.31]
More than high school	.99	.84		2.70	[.53	13.87]	-1.34	1.34		.26	[.02	3.63]
Subjective health (1: very bad - 5: very good)	.06	.24		1.07	[.67	1.70]	-.02	.20		.98	[.67	1.44]
Household income (1: far below average - 5: far above average)	-.07	.28		.93	[.53	1.62]	.61	.29	*	1.84	[1.05	3.23]
Living alone (1: yes, 0: no)	-.33	.82		.72	[.14	3.56]	.28	.44		1.33	[.56	3.12]
Employed (1: yes, 0: no)	.45	.55		1.57	[.54	4.62]	.26	.53		1.30	[.46	3.68]
Years living in the community	-.08	.18		.92	[.65	1.31]	-.11	.18		.90	[.63	1.28]
Wish to make contributions towards society	.23	.18		1.26	[.88	1.79]	.08	.14		1.09	[.83	1.42]
Number of people one contacts daily (family/relatives)	-.43	.29		.65	[.37	1.14]	.17	.25		1.19	[.73	1.93]
Number of people one contacts daily (other than family/relatives)	1.05	.28	***	2.87	[1.67	4.93]	.40	.19	*	1.49	[1.03	2.15]
With how many neighbors are you on greeting terms?	-.06	.22		.95	[.61	1.47]	-.19	.21		.83	[.55	1.23]
Level of urbanization (1: rural - 5: urban)	-.14	.26		.87	[.52	1.46]	-.14	.20		.87	[.58	1.29]
-2log likelihood	318.7						448.0					
df	39						39					
Pseudo-R^2 Nagelkerke	.42						.30					

Reference category is 'inactive type'.

Note. * $p < .05$, ** $p < .01$, *** $p < .001$.
